# Probing the Rare Biosphere of the North-West Mediterranean Sea: An Experiment with High Sequencing Effort

**DOI:** 10.1371/journal.pone.0159195

**Published:** 2016-07-21

**Authors:** Bibiana G. Crespo, Philip J. Wallhead, Ramiro Logares, Carlos Pedrós-Alió

**Affiliations:** 1 Institut de Ciències del Mar, Consejo Superior de Investigaciones Científicas (ICM-CSIC), Passeig Marítim de la Barceloneta, 37–49, 08003, Barcelona, Spain; 2 Norwegian Institute for Water Research (NIVA), Thormøhlens gate 53D, N-5006 Bergen, Norway; Estacion Experimental del Zaidin - CSIC, SPAIN

## Abstract

High-throughput sequencing (HTS) techniques have suggested the existence of a wealth of species with very low relative abundance: the rare biosphere. We attempted to exhaustively map this rare biosphere in two water samples by performing an exceptionally deep pyrosequencing analysis (~500,000 final reads per sample). Species data were derived by a 97% identity criterion and various parametric distributions were fitted to the observed counts. Using the best-fitting Sichel distribution we estimate a total species richness of 1,568–1,669 (95% Credible Interval) and 5,027–5,196 for surface and deep water samples respectively, implying that 84–89% of the total richness in those two samples was sequenced, and we predict that a quadrupling of the present sequencing effort would suffice to observe 90% of the total richness in both samples. Comparing the HTS results with a culturing approach we found that most of the cultured taxa were not obtained by HTS, despite the high sequencing effort. Culturing therefore remains a useful tool for uncovering marine bacterial diversity, in addition to its other uses for studying the ecology of marine bacteria.

## Introduction

The question of how many species of living beings there are on Earth has intrigued ecologists and evolutionary scientists for decades [1,2]. One of the most recent estimates suggested around 8.7 million species, but this excluded bacteria and archaea [3]. The International Census of Marine Microbes set out to map the diversity of microbes in the oceans with high-throughput sequencing (HTS) techniques [4] but a global estimate of the number of species was not attempted. Some estimates for marine bacteria range from 10^3^ to 10^6^ species based on different assumptions [5,6]. Such a range of values, spanning several orders of magnitude, shows that we are far from an accurate estimate.

Traditionally, bacteria were isolated in pure culture and then characterized biochemically and genetically until a new species could be formally described. It was realized that the bacteria able to grow in culture media were a small fraction of the bacterial cells that could be directly counted on a filter, a discrepancy named the “great plate count anomaly” [7]. Different studies estimated that only about 1% of the cells in natural waters could be cultivated [8,9]. Moreover, most of the cells in pure cultures were not the abundant ones in nature.

After the application of molecular cloning to natural systems [8,10] a wealth of new taxa were found and, this time, they were the abundant ones in the oceans [8,11]. The drawback was that a sequence of the 16S rRNA gene did not provide much information about the physiology of the organism. Further, the bacterial species obtained in culture were mostly different to those obtained in clone libraries. Molecular methods would retrieve many sequences from the abundant organisms but missed the rare ones, and only occasionally a rare clone was found. This was a simple consequence of the fact that natural assemblages are formed by a few taxa at very high abundance and many taxa in very low abundance (see Fig 2 in [12]). Primers for clone libraries would hybridize with the most abundant sequences over and over again, yielding only a small fraction of the community available to cloning and sequencing. Today, however, the development of high-throughput techniques and their application to natural microbial communities [13] raises the prospect of a quasi-exhaustive mapping of marine microbial diversity.

The study of microbial communities with HTS has revealed a wealth of novel sequences found in very low abundance, suggesting the existence of a "rare biosphere" of microbial populations [13] which has been the subject of several investigations [14–19]. Today, studies of microbial diversity are performed almost exclusively with such HTS techniques, yet culturing may still be considered indispensable [20–22], especially if the aim is to explore the rare biosphere. [21] compared the outputs of a pyrosequencing (~ 2,000 sequences per sample) analysis of the bacteria collected from a soil sample and the isolates cultured from the same sample. They found that 61% of the cultured bacterial species were not present in the pyrosequencing dataset, demonstrating that culturing can be complementary to sequencing for mapping microbial diversity.

In this study we aim to test whether or not contemporary HTS capabilities are sufficient to (i) uncover most of the bacterial richness in a marine water sample, and (ii) sequence all the taxa that are observed in culture. We address these questions by combining high sequencing effort (~500,000 final reads per sample) with advanced parametric statistical analyses, allowing us to obtain unusually well constrained estimates of total species richness and required sequencing effort for two marine water samples.

## Materials and Methods

### Study Area and Sampling

Samples were taken at Station D, an open sea station at 40°52’N and 02°47’E (Table 1, and [23]) in the NW Mediterranean Sea, during the SUMMER cruise between 13^th^ and 22^nd^ of September 2011 on board the RV “García del Cid”. The surface sample was taken at 5 m on the 15^th^ of September and the bottom sample was collected at 2000 m depth on the 17^th^ of September. No specific permissions were required for these locations/activities that were within Spanish waters. This field study did not involved endangered or protected species.

**Table 1:** Summary of location and depth (m) of samples, total sequences before cleaning (Raw Reads) and after cleaning (Final Reads), observed richness (S_obs_) computed as the total number of Operational Taxonomic Units (OTUs) clustered at 97% identity, and the percentage of singletons. Total richness (S) was estimated using the Chao1 lower bound estimator [39] and using the Sichel distribution fitted to the count frequency data by the Bayesian method of [33] and selected from four alternative candidate models using the Deviance Information Criterion (DIC). Using the Sichel distribution, point estimates and 95% credible intervals (CIs) for S were obtained from the mean and (2.5%, 97.5%) quantiles of the posterior distribution sampled 150000 times by Markov Chain Monte Carlo (after a burn-in period of 100000 samples, see 33). The Required Sequencing Effort (RSE) to sequence 90% of the total richness was predicted by hierarchical simulation (see Materials and methods) and is quoted in terms of the number of final reads and as a multiple of the present sequencing effort. Point estimates and 95% prediction intervals (PIs) for RSE were calculated as the mean and (2.5%, 97.5%) quantiles from a set of 80 simulations using the Sichel distribution.

	Surface	Bottom
Lat, Long	40°52’N, 02°47’E	40°52’N, 02°47’E
Depth (m)	5	2000
Raw Reads	713076	970346
Final Reads	500262	574960
OTUs 97% identity (S_obs_)	1400	4460
Singletons (% OTUs)	17.86	17.2
Total richness (S):		
Chao1 point estimate	1646	5031
Sichel point estimate	1615	5109
Sichel 95% CI	1568–1669	5027–5196
Required Sequencing Effort (RSE) for 90% of total richness:		
Sichel point prediction (final reads)	0.9x10^6^	1.2x10^6^
Sichel 95% PI (final reads)	(0.3–2.2)x10^6^	(0.6–1.9)x10^6^
Sichel point prediction / present effort	1.8	2
Sichel 95% PI / present effort	0.6–4.3	1.0–3.2

Sampling was carried out with Niskin bottles mounted on a rosette with a conductivity-temperature-depth (CTD) profiler. Water was prefiltered through a 200 μm mesh and processed on board. To collect microbial biomass, 5–15 L of sea-water were prefiltered through a 3 μm pore size Durapore filter (Millipore, Cork, Ireland) and free-living bacterial biomass was collected on a 0.22 μm pore size Sterivex filter (Durapore, Millipore). The samples were filtered in succession using a peristaltic pump. The 0.22 μm pore size Sterivex unit was filled with 1.8 ml of lysis buffer (40 mM EDTA, 50 mM Tris-HCl, 0.75 M sucrose) and stored at –80°C. DNA was extracted by a standard protocol using phenol/chloroform (details in [24]). The same amount of DNA for both samples was sequenced.

### 454-Pyrosequencing (HTS) and Noise Removal

Purified DNA samples were submitted to the Research and Testing Laboratory (Lubbock, Texas, USA). Bacterial diversity was assessed by tag-pyrosequencing of the V1-V3 regions of the 16S rRNA gene with the Roche 454 Titanium platform and manufacturer protocols (454 Life Science). The hypervariable regions were amplified using Primers 28F (5’-GAGTTTGATCNTGGCTCAG) and 519R (5’-GTNTTACNGCGGCKGCTG). Approximately 400 base-pairs (bp) were obtained for each read. PCR and subsequent sequencing are described in [25]. 713,076 and 970,346 reads were retrieved from the surface and bottom samples respectively (Table 1). These data have been deposited in EMBL with accession number PRJEB9061.

The raw tag-sequences (reads) were processed using QIIME [26]. Briefly, to reduce sequencing errors and their effects, the multiplexed reads were first trimmed, quality-filtered and assigned to the samples (surface or bottom). The filtering criteria included a perfect match to the sequence barcode and primer, at least 400 bp in length, and an average quality score (phred) of 28 within sliding windows of 50bp. The amount of erroneous sequences was further reduced using Denoiser [27]. The sequences were then clustered into Operational Taxonomic Units (OTUs) based on the relatedness of the sequences (97% identity) using UCLUST, version 1.1.579 [28]. A representative sequence from each OTU was selected as the first cluster seed chosen by UCLUST. Chimeras were checked with ChimeraSlayer implemented in Mothur [29]. Taxonomy was then assigned with QIIME by searching the representative sequences of each OTU against the SILVA 16S/18S rRNA gene non-redundant reference dataset (SSU Ref 108 NR) [30] using the Basic Local Alignment Search Tool (BLAST) and an e-value of 0.03. Any OTUs not identified by these criteria were removed from the output fasta file, since we could not be sure that they corresponded to bacterial species. Chimera, chloroplast, eukarya and archaea sequences were also removed. The remaining final reads were used to construct a table of identified bacterial OTUs and their corresponding abundances for each water sample.

### Isolation of Bacteria in Cultures

Isolates from the surface sample were taken on board from a Niskin bottle closed at the surface. Isolates were obtained by plating 100 μl of undiluted and 10x diluted sea-water from the surface sample, in triplicates, onto modified ZoBell agar plates (i.e. 5 g peptone, 1 g yeast extract and 15 g agar in 1 l of 0.2 μm filtered 75% sea water). Agar plates were incubated at *in situ* temperature (~20°C), in the dark, for 14 days. 326 bacterial colonies were selected and the cultures were subsequently purified by re-isolation three times in a month. Next, the isolates were grown at 20°C on the same liquid medium and stored at -80°C with 25% (v/v) glycerol. 200 μl of these cultures were placed in 96 well plates, diluted 1:4 and heated (95°C, 10 min) to cause cell lysis, so that the available DNA could be used as a template in Polymerase Chain Reactions (PCR). As many different species as possible from the 326 isolates were selected by PCR with Taq polymerase (Boehringer-Mannheim) of the Internal Transcribed Spacer (ITS) using primers ITS-F (5’-GTCGTAACAAGGTAGCCGTA) and ITS-R (5’-GCCAAGGCATCCACC) and the following thermal conditions: 94°C for 2 min, then 32 cycles of 94°C for 15 sec, 55°C for 30 sec, 72°C for 3 min, followed by one cycle of 72°C for 4 min and 4°C on hold. ITS length is species specific and therefore allows us to differentiate the isolates [31,32]. According to their different ITS patterns, 148 isolates out of 326 were chosen conservatively i.e. including some replicates or isolates with visually similar ITS pattern to prevent excluding any different species. The 16S rRNA genes of the chosen isolates were amplified using bacterial 16S rRNA gene primers 27F (5'-AGAGTTTGATCMTGGCTCAG) and 1492R (5'-GTTTACCTTGTTACGACTT) in the following thermal conditions: 94°C for 5 min, then 30 cycles of 94°C for 1 min, 55°C for 1 min, 72°C for 2 min, followed by one cycle of 72°C for 10 min and 4°C on hold. Nearly the full-length 16S rRNA gene (approx. 1300 bp) was sequenced using Sanger sequencing in GENOSCREEN (Lille Cedex, France). Taxonomy of the isolates was assigned by BLAST searches in the National Center for Biotechnology Information (NCBI) website, and any unidentified isolates were filtered from our results. The 16S rRNA gene sequences have been deposited in EMBL with accession numbers LN845965 to LN846112.

### Richness and Sequencing Effort Estimates from the 454 Pyrosequencing (HTS) Data

Observed species richness (S_obs_) was computed as the total number of identified bacterial OTUs from sequencing each DNA sample. We define the total species richness (S) as the total number of identifiable bacterial OTUs in the water sample i.e. the richness that would be observed if we were able to sequence all of the DNA in the water sample. It was assumed that the sequenced DNA could be considered a random sample from a very much larger total quantity of DNA in the water sample. Total species richness was estimated by fitting a parametric distribution to the count data obtained by sequencing, following the Bayesian Markov-Chain Monte Carlo (MCMC) method of [33]. We fitted four distributions: the Poisson log-normal, the Poisson log-Student, the Poisson inverse Gaussian, and the Poisson generalized inverse Gaussian (Sichel distribution). The best-approximating distribution for each sample was chosen using the Deviance Information Criterion (DIC [34]), which for our fits was almost identical to Akaike’s Information Criterion (AICc [35]; Table A in S1 File). S was then estimated as the posterior mean value of the corresponding Bayesian parameter under the selected model, and 95% credible intervals (CIs, Bayesian equivalent of confidence intervals) were taken from the 2.5% and 97.5% quantiles of the posterior distribution. Note that by this method the total richness S is included in the likelihood function and estimated jointly with the two or three parameters describing the taxon abundance distribution, thus facilitating uncertainty calculations [36,37]. Also, the Bayesian MCMC approach appears to mitigate the problem of trapping in local maxima which can compromise the calculation of maximum likelihood estimates [38].

We also predicted the required sequencing effort (RSE) to observe 90% of the total species richness in a hypothetical repeat DNA sample from the same water sample. Higher percentages were not considered because due to uncertainties in the estimates they could not be meaningfully constrained. RSE was predicted by simulating a set of 80 repeat sequences using the selected model and sampling from the posterior parameter distribution, then taking the mean RSE and percentiles (2.5%, 97.5%) over the set as point predictions and 95% prediction intervals (PIs) respectively. For each of 80 simulations, we simulated a random sequence of 10N individual species labels, where N is the present sequencing effort. This was done by: 1) sampling a set of parameter values (including total richness S) from the posterior distribution, 2) sampling relative abundances (proportions in the water sample) from the taxon abundance distribution given the parameter values, 3) sampling species counts (from hypothetical sequencing) using the multinomial distribution given the relative abundances and the total number of individuals 10N, and 4) converting the species counts into a randomly-ordered sequence of final reads. The simulated RSE was then identified as the read (tag) index for which the number of species observed earlier in the sequence first exceeded 90% of the simulated total richness (S). Model selection uncertainty [35] was not accounted for in the PIs for RSE nor in the CIs for S; however, the only model with comparable DIC to the best-approximating model (to within 12 units of DIC or AICc) was merely a special case of the best-fitting model (Poisson inverse Gaussian vs. Sichel distribution, see Table A in S1 File) so the neglected uncertainty was likely small.

These simulations and others using the non-selected distributions were also used to test the performance of various simpler and faster methods to predict S and RSE, including several nonparametric methods [39–48] and a semiparametric method whereby multiple saturating functions are fitted to the collector’s curve and the lowest-AICc function is used for prediction (Tables B and C in S1 File; [49,50]). Unfortunately, none of these faster methods showed robust performance over all simulations (Table D in S1 File; [33,51]). Herein, we report only the Chao1 estimator for S [39] because it is widely quoted and thus useful for comparison with other studies. Rank-abundance curves of the isolated cultures and the HTS data were plotted using “BiodiversityR” [52] and collector’s curves with confidence intervals were computed using “iNEXT” [47,53].

### Comparison of 454-Pyrosequencing (HTS) Reads and Cultured Isolates

Isolates and 454 tag-sequences were compared by running BLASTn locally. The isolate sequences were searched for in the HTS datasets and vice versa, and only the reciprocal matches between these two searches were considered. The output was filtered using R [54], requiring 99% of identical nucleotide matches, ≥75% coverage of the isolate sequence, and a bit-score higher than 100. In all cases the e-value was lower than 0.0001.

The primers used for Sanger sequencing of the isolates and those used for the pyrosequencing of the environmental DNA were different, which could cause different biases that could prevent the detection of the cultures in the HTS dataset. To investigate this, the sequences of the isolates and the sequences of the pyrosequencing primers were multiple-aligned using the software Geneious pro 3.5.4 [55]. This analysis showed that the HTS primers hybridized with the sequences of all the isolates, leaving us no reason to suspect a bias due to different primers.

## Results

### Pyrosequencing (HTS) Dataset

**Fig 1:**
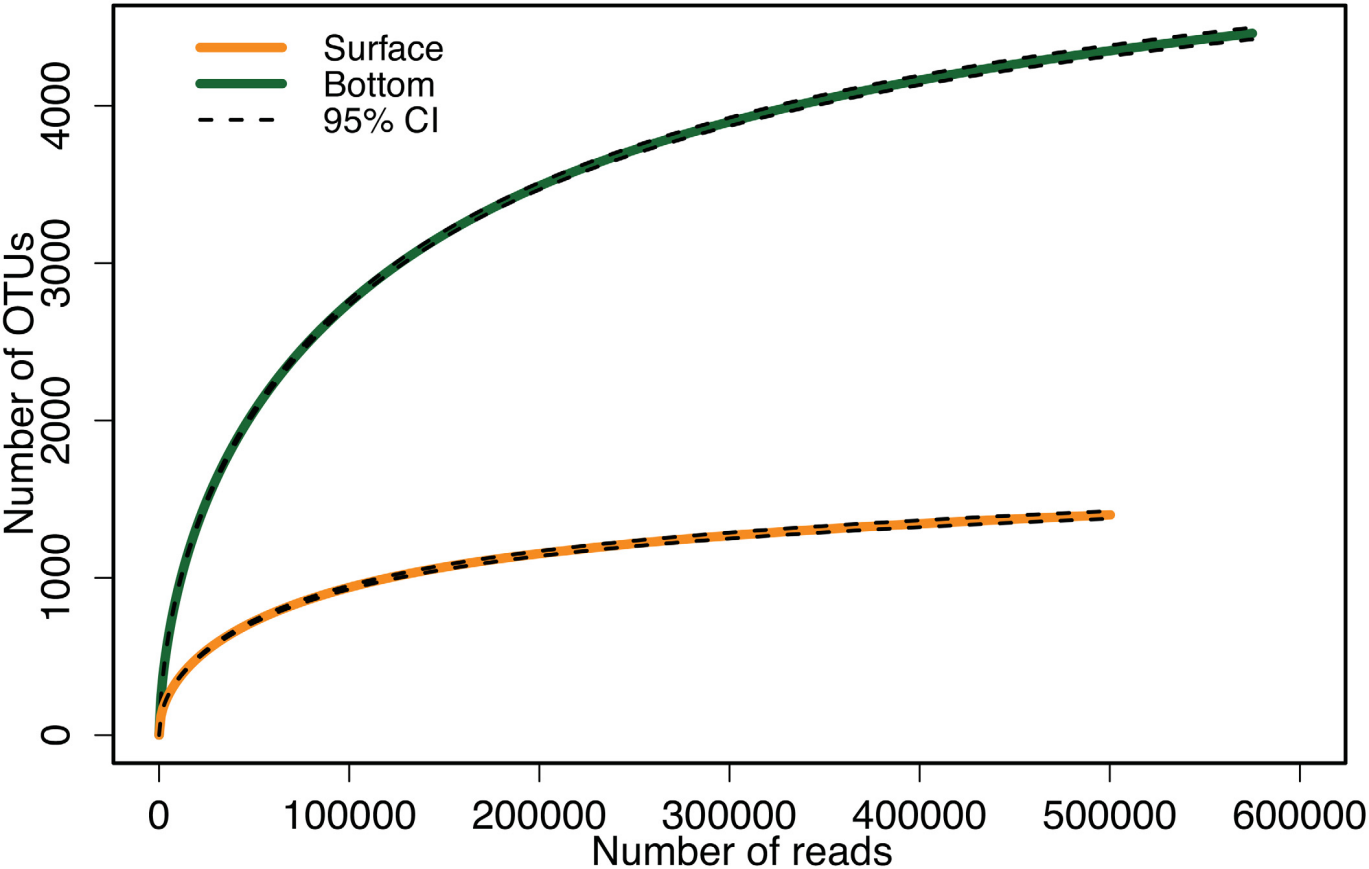
OTU collector’s curves of the surface (orange line) and bottom (green line) samples. Black dashed lines indicate the 95% confidence intervals (95% CI).

Observed richness (S_obs_) was much higher in the bottom (4,460) than in the surface (1,400) sample (Table 1). In both samples only ~17% of the Operational Taxonomic Units (OTUs) were singletons (an OTU represented by a single sequence) (Table 1). Collector's curves suggested that the bottom sample would be richer for a broad range of lesser, equal sequencing efforts and that S_obs_ was approaching asymptotic values for both samples (Fig 1).

Among the four candidate parametric distributions fitted to the count data, the Sichel distribution was the best approximating model (lowest Deviance Information Criterion, DIC and Akaike’s Information Criterion, AICc) for both samples (Table A in S1 File). The goodness-of-fit of this distribution is illustrated in Fig A in S1 File. The fitted frequencies at moderately low counts may suggest some room for improvement, but overall for the counts in the range 1–100 shown in Fig A in S1 File it appears that the model gives an adequate fit. Using the Sichel distribution, the total water sample richness was estimated as 1,568–1,669 (95% Credible Interval, CI) and 5,027–5,196 for surface and deep samples respectively, suggesting that 84–89% and 86–89% of the total richness was observed by sequencing. By simulating from this distribution we predict that 0.6–4.3 (95% Prediction Interval, PI) and 1.0–3.2 times the present sequencing effort would suffice to observe 90% of the total richness in the surface and bottom water samples respectively (Table 1).

**Fig 2:**
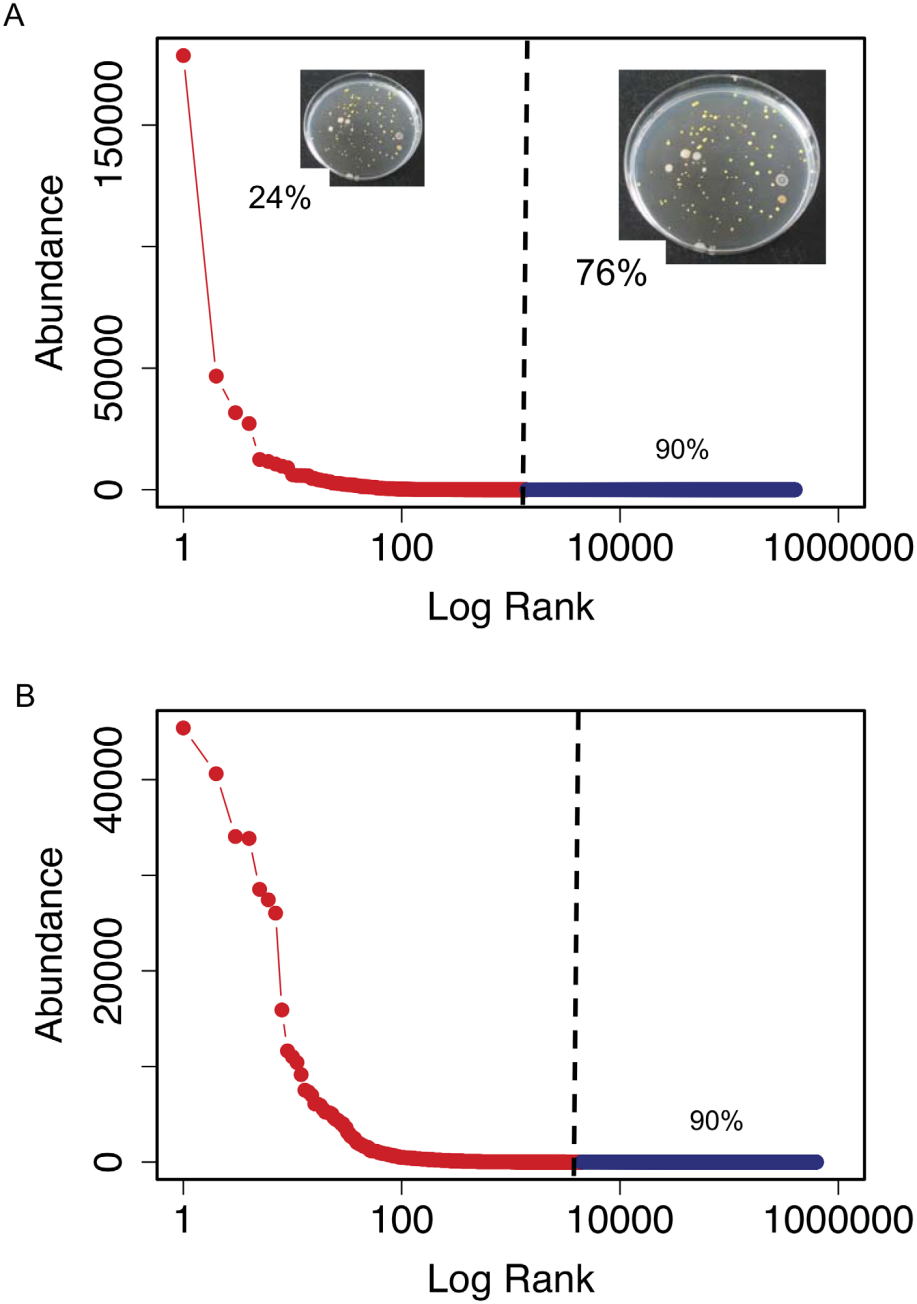
Rank-abundance plots of surface (A) and bottom (B) samples. The red line is the rank-abundance plot calculated with the actual data. The dark blue line shows the estimates of the sequencing effort necessary to retrieve 90% of the total richness calculated by simulation from the best-approximating Sichel distribution (posterior mean estimate). The vertical black line separates the real data (left) from the estimates (right). In (A) the percentage of cultured isolates found in the 454-pyrosequencing dataset is indicated at the left side of the black vertical line. The percentage of cultured isolates not found in the 454-pyrosequencing dataset, and that would presumably be found by increasing the sequencing effort, is indicated at the right of the black vertical line. Insert pictures show some of the bacterial cultures grown from the surface sample. Font size and pictures are scaled according to the percentage of cultured isolates found or not found in the 454-pyrosequencing dataset.

Rank-abundance curves (Fig 2) showed that the bacterial assemblages from both samples were characterized by few abundant and many rare OTUs. The most abundant OTU was more abundant in the surface than in the bottom sample. The abundance of the most abundant OTU in the bottom sample was close to the abundance of the second most abundant OTU in the surface sample.

### Culture Collection

**Fig 3:**
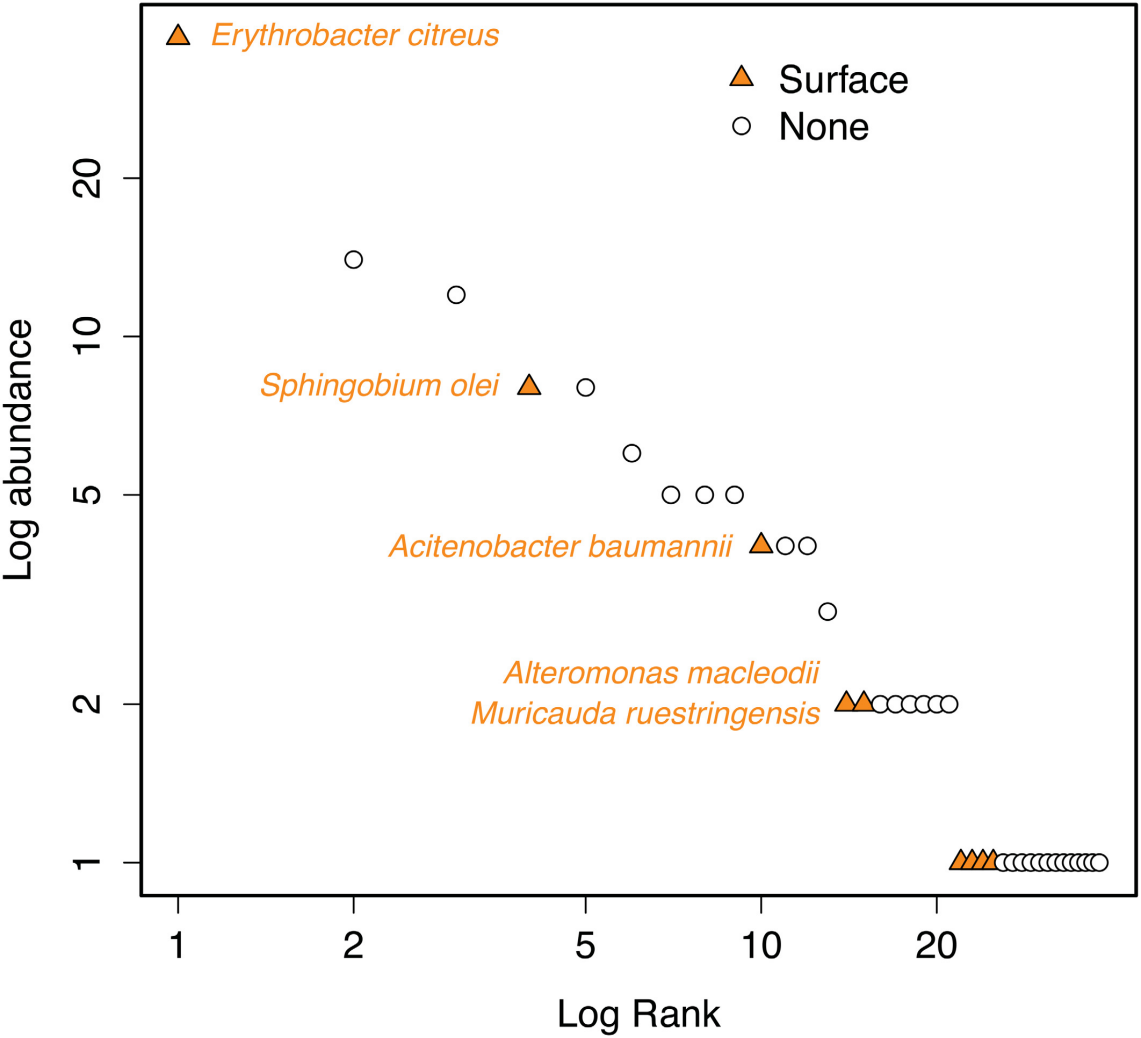
Rank-abundance plot of the 38 isolated bacterial species. The orange triangles indicate the cultured isolates found in the 454-pyrosequencing (HTS) dataset and the white circles indicate the cultures that were not found in the HTS dataset. The isolated bacterial species are listed in Tables 2 and 3.

Bacterial isolation from the sample collected at the surface retrieved 148 cultures belonging to 38 different species. The most frequent bacterium in the collection was *Erythrobacter citreus*, isolated 37 times, while 17 species were isolated only once. A rank abundance plot of the 38 species is shown in Fig 3. The isolates belonged to the phyla Actinobacteria (4 isolates), Bacteroidetes (4 isolates) and Firmicutes (2 isolates) and to the Proteobacteria classes Alpha-proteobacteria (18 isolates) and Gamma-proteobacteria (10 isolates). The names of all the isolates are shown in Tables 2 and 3.

**Table 2:** Cultured isolates with matching HTS sequences. Columns show the isolates’ closest relatives according to the BLAST results, the percentage of identity with the BLAST reference strain (identity BLAST), the GenBank accession number of the BLAST reference strain, the number of HTS reads matching the isolate sequences in the surface sample (Reads in Surface), the percentage of the total HTS reads in the surface sample represented by the isolate sequences (% Surface), and the number of isolates of each taxa sequenced. Abbreviations are: Actino (Actinobacteria), Bact (Bacteroidetes), Alpha-P (Alpha-Proteobacetria) and Gamma-P (Gamma-Proteobacteria).

Isolates’ closest relative	Identity BLAST	GenBank accession number	Reads in Surface	% Surface	Number of isolates
*Uncultured Brevundimonas* sp. (Alpha-P)	99.90%	JX047099	76	1.52x10^-2^	1
*Alteromonas macleodii* str. 'Balearic Sea AD45' (Gamma-P)	100%	CP003873	40	8.00x10^-3^	2
*Sphingobium olei* (Alpha-P)	100%	HQ398416	34	6.80x10^-3^	8
*Erythrobacter citreus* (Alpha-P)	100%	EU440970	31	6.20x10^-3^	37
*Citromicrobium* sp.(Alpha-P)	100%	HQ871848	22	4.40x10^-3^	1
*Acinetobacter baumannii* (Gamma-P)	100%	JX966437	16	3.20x10^-3^	4
*Bizionia* sp. (Bact)	100%	EU143366	13	2.60x10^-3^	1
*Muricauda ruestringensis* (Bact)	99%	JN791391	4	8.00x10^-4^	2
*Microbacterium jejuense* (Actino)	100%	AM778450	1	2.00x10^-4^	1

**Table 3:** Cultured isolates without matching HTS sequences. Columns show the isolates' closest relatives according to the BLAST results, the % of identity with the BLAST reference strain (identity BLAST), the GenBank accession number of the BLAST reference strain and the number of isolates of each taxa sequenced. Abbreviations are: Actino (Actinobacteria), Bact (Bacteroidetes), Firm (Firmicutes), Alpha-P (Alpha-Proteobacetria) and Gamma-P (Gamma-Proteobacteria).

Isolates’ closest relative	Identity BLAST	GenBank accession number	Number of isolates
*Microbacterium aquimaris* (Actino)	99.60%	HQ009858	14
*Thalassospira* sp. (Alpha-P)	100%	EU440837	12
*Fulvimarina pelagi* (Alpha-P)	96%	HQ622550	8
*Alcanivorax* sp. (Gamma-P)	99.70%	AB681671	6
*Devosia subaequoris* (Alpha-P)	100%	JQ844475	5
*Halomonas aquamarina* (Gamma-P)	100%	AB681582	5
*Marinobacter flavimaris* (Gamma-P)	100%	AB617558	5
*Alterierythrobacter* sp. (Alpha-P)	100%	FM177586	4
*Alteromonas macleodii* (Gamma-P)	99.90%	CP003917	4
*Erythrobacter* sp. (Alpha-P)	100%	AB429073	3
*Bacillus horikoshii* (Firm)	100%	JQ904719	2
*Brevundimonas* sp. (Alpha-P)	99.90%	HQ830182	2
*Devosia hwasunensis* (Alpha-P)	99%	HQ697727	2
*Idiomarina seosinensis* (Gamma-P)	99.90%	EU440964	2
Rhizobiales family (Alpha-P)	96%	HQ622550	2
*Roseivirga spongicola* (Bact)	99.80%	NR043531	2
*Arthrobacter oxydans* (Actino)	100%	EU086823	1
*Bacillus* sp. (Firm)	100%	AM950311	1
*Emticicia* sp. (Bact)	100%	JX426065	1
*Halomonas* sp. (Gamma-P)	100%	HE586874	1
*Marinobacter hydrocarbonoclasticus* (Gamma-P)	100%	JQ799097	1
*Nitratireductor* sp. (Alpha-P)	99.90%	AM981316	1
*Nocardioides marinus* (Actino)	99.90%	NR043787	1
*Pseudomonas* sp. (Gamma-P)	99.90%	JN244973	1
*Sphingobium yanoikuyae* (Alpha-P)	99.90%	DQ659593	1
*Thalassospira permensis* (Alpha-P)	99.90%	FJ860275	1
Alphaproteobacterium	99.80%	AY515421	1
*Martelella mediterranea* (Alpha-P)	99.80%	EU440955	1
Uncultured *Nitratireductor* sp. (Alpha-P)	99.70%	AM981316	1

### Comparison of Isolates and 454 Tag-Sequences

Only 9 (24%) of the 38 different isolated species were found in the HTS dataset: 1 Actinobacteria, 2 Bacteroidetes, 4 Alpha-proteobacteria and 2 Gamma-proteobacteria isolates (Fig 3, Table 2). Almost all of the 454 tag-sequences that matched the sequences from the cultured isolates belonged to OTUs with low abundance in the HTS dataset (<1% of the total reads).

## Discussion

### Estimates of Species Richness

In a previous study [56] pyrosequencing of the V6 region of the 16S rRNA gene was used to estimate richness of the bacterial assemblages in the NW Mediterranean Sea, at the same location and month as the present study but during a different year. Around 20,000 final reads were obtained per sample and 632 and 2,065 OTUs were observed in surface and deep samples respectively. It is well known that the number of new taxa retrieved increases with sample size and sampling effort [57–59] and that a large part of the diversity may remain hidden due to sampling limitations [60], especially in microbial ecology [61]. In the present study, we took advantage of growing pyrosequencing capabilities to increase the sequencing depth (to around 500,000 final reads per sample) in an attempt to achieve realistic estimates of the whole bacterial richness in our samples.

The resulting collector’s curves appear to be approaching asymptotic values (Fig 1) and the reduced percentage of singletons (~17% vs. 40%–60% in [56]) suggests an improved coverage of the bacterial community. However, the order of magnitude of the Chao1 estimates of total richness are consistent with the earlier study (1,646 and 5,031 here vs. 1,289 and 4,156 in [56]), and our present Chao1 estimates agree with the 95% CIs from the best-approximating Sichel distribution (see Table 1). Also, if we use the collector's curve in Fig 1 to predict the observed species richness in the present samples at the sequencing effort of the earlier study (20,000 final reads) we obtain S_obs_ of (487, 1330) in surface and bottom samples respectively, which is not too far from the numbers observed in the previous study (632, 2065).

In the surface sample, the most abundant OTU contributed a very large fraction of the total reads (36%), raising concerns that this may have caused fewer OTUs to be uncovered and forced the richness to appear lower. However, if this taxon is excluded from the analysis, the main effect on the collector’s curves is to decrease the total number of reads for the surface sample by 36%, and the bottom sample is still clearly richer at this lower level of effort (Fig 1). We also reran the Sichel fit to the surface data with this OTU excluded and obtained a negligible change (1 species) in the estimated total richness (Table A in S1 File). The number of OTUs observed in both samples in this study are consistent with numbers estimated by other authors for the upper ocean [33,56,62,63] and the deep ocean [64]. 56 and 64 also found higher richness in the bottom than in the surface waters.

The estimates of total species richness suggest that our sequencing effort of ~500,000 final reads per sample was quasi-exhaustive, yielding 84–89% and 86–89% of the total richness in surface and bottom samples respectively. For comparison, the collector's curve (Fig 1) predicts that the lower effort of 20,000 final reads used in [56] would have yielded only around 29% and 26% of the total richness in surface and bottom samples respectively. This lower effort is clearly inadequate for studying the rare biosphere, although it may have been adequate to establish the higher richness of the bottom sample in this particular case (see Fig 1). Simulations with the best-fitting Sichel distribution suggested that a further factor of four increase in sequencing effort (~2 million final reads) would be adequate to sequence 90% of the total identifiable bacterial species richness in the present water samples. Of course, such predictions will be gradually invalidated as more and more species are discovered, increasing the number of identifiable OTUs. However, the current rapid development of sequencing technologies raises the prospect of orders of magnitude increases in HTS final reads in the near future, suggesting that the 90% threshold may soon be regularly crossed.

It is worth noting that the development of HTS capability needs to be matched by the development of statistical estimation methods that are adequate for the microbial setting. Current approaches to richness estimation are dominated by simple nonparametric methods that have been developed and refined principally for applications outside of microbial ecology, for communities that do not exhibit such extreme variations in relative abundance between species (e.g. [39,45,61]). Although the Chao1 estimator gave total richness estimates that were consistent with the Sichel estimates for our particular two water samples (Table 1), our simulation tests showed that the Chao1 estimator can give strongly biased estimates when data are simulated using some of the candidate parametric distributions fitted to the present data (Table D in S1 File). Large biases were also observed in simulation tests of the bias-corrected "iChao" estimator [48], the "ACE" estimators proposed for highly uneven communities [40,41], and a semi-parametric approach based on fitting saturating functions to the collector's curves (Table D in S1 File). Similar findings have been reported elsewhere [33,51] and suggest that alternative methods may be required for microbial ecology. We found that the Bayesian parametric estimation method of [33] resulted in satisfactory fits to the observed count-frequency data using the Sichel distribution (Fig A in S1 File). The Bayesian method was itself too computationally intensive to allow testing by simulation, but conventional wisdom suggests that parametric estimates will generally perform well in cases where the parametric model is a close approximation to the true abundance distribution [51]. A limitation here is the small number of candidate taxon abundance distributions (four) that the Bayesian MCMC software currently allows the user to fit, and we hope that this library will be expanded in the future to minimize the risk of estimator bias.

### Comparison of HTS and Culture Isolation

The current power of massive parallel sequencing allows us to probe the rare biosphere [16,17,19], but culturing (isolation) is an alternative avenue to explore it [12,21]. Comparing these two approaches we found that isolation retrieved some of the rarest taxa (Table 2) and that only 24% of the isolates were found in the HTS data (Fig 2A).

The observed and estimated total values of richness can be used to estimate the probability that a species chosen at random from the total list of species is retrieved by the present HTS effort. This probability is S_obs_/S ≈ 0.87 for the surface sample, so if the 38 cultured species could be considered randomly chosen, we would expect to retrieve 33 of them by HTS. Given this probability, the fact that we retrieved only 9 (24%) is highly significant (P < 10^−17^ from binomial test; P < 1/3001 from simulation test accounting for uncertainty in the retrieval probability, text in S1 File). The cultured species are apparently less represented in the HTS datasets than would be random selections from the lists of all species in the water samples.

We see three possible reasons for this discrepancy. First, there may have been a bias due to different PCR and DNA amplification of the different sequencing techniques used to identify species in HTS vs. in culture [65,66]. However, when tested *in silico* (see Materials and Methods), the primers used for pyrosequencing (HTS) covered the whole diversity captured by the primers used for Sanger sequencing of the isolates, suggesting no significant bias due to different sequencing techniques.

Second, since the cultures were isolated from the whole water sample while the HTS data were obtained from the 0.2–3 μm fraction, some species attached to larger particles may have been excluded from the HTS datasets. However, 18 of the 38 cultured species are expected to be free-living bacteria since they belong to the Alpha-proteobacteria class [63,67] and should therefore be present in the 0.2–3 μm fraction used for HTS. If the comparison is restricted to this class we find that only 4 out of 18 isolates are retrieved in the surface HTS dataset, which is still a highly significant deficit (P < 10^−9^, binomial test; P < 1/3001, simulation test).

A third possible reason is that the special environment imposed by culturing may favour certain species that are generally less successful in natural oceanic conditions, and consequently too rare to retrieve with the present HTS effort. The process of culturing might in this sense “select for the losers” in the natural environment. However, if this were a consistent effect, we would expect the few isolated species that are retrieved by HTS to have anomalously low HTS abundance, but this is not in fact observed (Table 2). The surface counts, while low in an absolute sense (<0.1% of total reads), are not low relative to a random sample from the observed or modelled count distributions (P > 0.05 from bootstrap and simulation tests on mean, median and maximum counts, text S1 File). The culturing process might therefore have selected for a few moderately rare species (Table 2) plus a larger number of extremely rare species that could not be retrieved with the present HTS effort (Table 3). Culturing conditions different from those used in this study would surely have yielded a different outcome. We can hypothesize that by varying the incubation conditions (light levels, oxygen, medium composition, etc.) the relative abundance of different target subsections of the bacterial community might be magnified, allowing their diversity to be mapped more efficiently than by HTS of natural water samples where the target relative abundances may be prohibitively low.

Our results suggest that, with the HTS capacity of 2012, culturing remains an important complementary tool for uncovering microbial diversity. Future improvements in HTS depth will eventually uncover the isolated bacteria, though perhaps only slowly. However, even if the whole bacterial diversity were mapped by HTS, culturing would remain essential for the study of marine bacterial communities, especially if the target is the rare biosphere [20]. Culturing provides complete genomes and allows the study of the physiology, metabolism and ecology of marine bacteria, yielding information that cannot be obtained by HTS alone [68].

The question remains to what extent our results can be generalized or extrapolated to larger spatiotemporal regions than those defined by the two 20L water samples from which the DNA samples were extracted. Recall that the sequenced DNA was assumed to be a random sample from a much larger total quantity of DNA in the water sample. If this is true, our estimates of total species richness should be valid for the total numbers of species in the water samples. To apply such an estimate to a larger "community" or "assemblage" we must assume that the total species inventory for this larger region is identical (or very similar) to the total species inventory for the corresponding water sample (note, however, we do not need to assume that the relative abundances for the larger region are similar to those of the water sample). The level of homogeneity or overlap of species inventories over an extended region/period could be investigated by analysing and comparing multiple water samples dispersed within this region/period. However, experimental designs that include replicated water samples must necessarily limit the investment in HTS effort per water sample, and are therefore less suited to investigating the limits of current HTS capabilities as is our aim herein. In the future we hope that it will become possible to analyse data from deeply-sequenced and replicated water samples, allowing quasi-exhaustive species inventories for extended marine bacterial communities to be derived with greater confidence.

To summarize, we return to the two questions posed in the Introduction. Regarding (i) the answer is Yes: current HTS capabilities *can* yield quasi-exhaustive mapping of bacterial species richness in a marine water sample. Deep HTS analyses allowed us to obtain collector’s curves that are approaching asymptotes, which to our knowledge has not been shown before for a marine bacterial assemblage in a single water sample. The sequencing depths required to do this—order of a million final reads—may be impractical at present for routine or replicated studies, but rapidly-developing sequencing technologies may soon alleviate this burden. Regarding (ii) the answer is No: currently feasible HTS depths appear to be still insufficient to retrieve all the species that may be isolated by culturing. We arrive at a perhaps surprising conclusion that culturing analysis can still be complementary to HTS even for the simple mapping of diversity by listing species present. Our study therefore confirms that HTS and culturing remain complementary strategies for probing the rare marine biosphere.

## Supporting Information

S1 File Text; Fig A and Tables A, B, C and D. (PDF)
