# Supplementary material for: Probing the Rare Biosphere of the North-West Mediterranean Sea: An Experiment with High Sequencing Effort
Source: PLoS One. 2016 Jul 21;11(7):e0159195. doi: 10.1371/journal.pone.0159195 (PMC4956085; doi:10.1371/journal.pone.0159195)
Supplement: S1 File — (PDF) [file pone.0159195.s001.pdf]

Probing the rare biosphere of the North-West Mediterranean Sea: an experiment with high sequencing effort

Bibiana G. Crespo, Philip J. Wallhead, Ramiro Logares and Carlos Pedrós-Alió

Supporting information

Text.

Discussion: Simulation tests on the number of isolates retrieved in pyrosequences

To simulate the number of isolates retrieved in pyrosequences, we simulated 3000 sets of species counts using the method described above for RSE calculations, but with the total number of reads fixed at the present sequencing effort N. For each set of simulated sequencing counts, 38 species were selected at random without replacement from the list of all S counts (including zeros), and the number of these with non-zero counts was recorded to give the simulated number retrieved by sequencing r_s. The simulation p-value for the actual number of species retrieved by sequencing r was then taken as $(1 + \#(r_s \leq r))/3001$ following Davison and Hinkley (1997).

Discussion: Simulation/bootstrap tests on the counts of isolates retrieved in pyrosequences

To simulate the counts of isolates retrieved in pyrosequences, we again simulated 3000 sets of species counts as described above, and this time randomly selected without replacement 9 species from the list of non-zero counts for each simulation. The mean, median, and maximum counts from this subset were recorded for each simulation, and p-

values were calculated as described above assuming lower-than-random count statistics as an alternative hypothesis.

These tests were also repeated using a bootstrap method, thus avoiding the need to assume a parametric distribution. To do this, a vector of 9 species counts was randomly resampled *with* replacement from the observed species count vector. This was repeated over 9999 bootstraps and bootstrap p-values were calculated as $(1 + \#(t_s \leq t))/10000$ or $(1 + \#(t_s \geq t))/10000$, again following Davison and Hinkley (1997).

References for the supporting information text:

1. Davison A, Hinkley D. Bootstrap methods and their applications. New York: Cambridge University Press; 1997.

Figure A. Goodness-of-fit of the best-approximating Sichel distribution to (A) surface and (B) bottom HTS datasets. Observed and predicted count frequencies (numbers of OTUs with a given sample abundance) are plotted against read counts (sample abundances) on a log-log scale. Goodness-of-fit is illustrated by the closeness of the predicted frequencies (posterior means, solid lines) to the observed frequencies (dots) as well as by the narrowness of the 95% prediction intervals (dashed lines) while still containing most of the data. The comparison is restricted to rare counts in the range 1–100 because these are likely the most important for estimating total richness and required sequencing effort, and because the computation of stable frequency prediction intervals for higher counts would require too many simulations (the intervals shown used 3000). The distributions were however fitted to the full range of observed count frequencies ($f_{1\text{–}178569}$ and $f_{1\text{–}45414}$ for surface and bottom samples respectively).

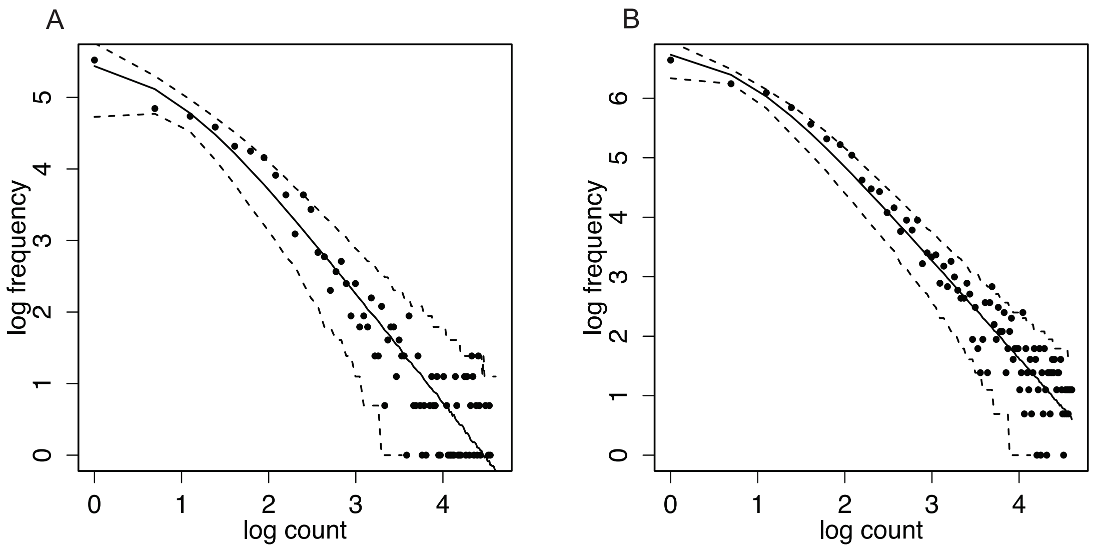

Table A. Four different compound Poisson distributions were fitted to the surface and bottom HTS data: the Poisson log-normal, the Poisson inverse Gaussian, the Poisson log-student, and the Poisson generalized inverse Gaussian (Sichel) distribution. As a robustness check we reran the Sichel fit for the surface sample excluding the counts of the most abundant species which, for this sample, was more than 3 times as abundant as the second most abundant species (see Surface*). The relative goodness-of-fit is assessed using Akaike's Information Criterion (AICc = $-2 \times \max(\log \text{likelihood}) + 2p + 2p(p+1)/(n-p-1)$, where p is the number of fitted parameters and n is the number of data; Hurvich and Tsai, 1989; Burnham and Anderson, 2002) and the deviance information criterion (DIC = $-2\times$posterior mean(log likelihood) + p; Spiegelhalter *et al.*, 2002; Quince *et al.*, 2008). For the robustness check the selection criteria are placed in square parentheses since these cannot be compared to other rows. We also show the total species richness estimates from maximum likelihood ($\hat{S}_{ML}$) as well as the posterior median ($\hat{S}_{50\%}$) and the 95% credible bounds ($\hat{S}_{2.5\%}$ and $\hat{S}_{97.5\%}$) from the Bayesian MCMC method (Quince *et al*. 2008).

Reference: Hurvich, C.M. and Tsai, C.-L. (1989) Regression and time series model selection in small samples. Biometrika 76: 297-307.

Distribution	No. fitted parameters *p*	Sample	min (-log lik)	AICc	DIC	$\hat{S}_{max.\ lik.}$	$\hat{S}_{posterior\ mean}$	$\hat{S}_{50\%}$	$\hat{S}_{2.5\%}$	$\hat{S}_{97.5\%}$
Log-normal	3	Surface	869.4	1744.8	1744.8	2449	2501	2488	2238	2819
Log-student	4	Surface	840.6	1689.1	1689.3	1869	1897	1891	1797	2027
Inverse	3	Surface	836.9	1679.8	1679.9	1644	1644	1643	1594	1702
Sichel	4	Surface	834.7	1677.4	1677.3	1618	1615	1614	1568	1669
Sichel	4	Surface*	[821.3]	[1650.7]	[1651]	1619	1616	1615	1568	1671
Log-normal	3	Bottom	1276.9	2559.8	2559.8	6843	6856	6850	6544	7199
Log-student	4	Bottom	1198.0	2404.1	2404.1	5850	5867	5863	5701	6055
Inverse	3	Bottom	1230.0	2466.0	2466.0	5352	5353	5352	5250	5463
Sichel	4	Bottom	1176.9	2361.8	2362.1	5118	5109	5108	5027	5196

Table B. Semiparametric functional fits to surface sample collector's curve data and corresponding estimates of total species richness. A set of 12 convex, saturating functions were fitted to the rarefied species accumulation curve, sampled at intervals of 1000 reads (hence 502 data points), using the nonlinear least squares function "nls" in R to estimate the parameters a, b etc. The absolute quality of the fits was measured using the generalized R2 values (defined for nonlinear fit as 1 - RSS/SSM, where RSS is the residual sum of squares and SSM is the sum of squares of the sample mean). The best-approximating model was selected as that which minimized Akaike's Information Criterion (AICc, in this case the Power Michaelis Menten (2) function was selected). The selected model was then used to estimate the total sample richness S as the asymptotic value of the function at x = Inf (final column shows the estimates for all candidate functions). Required sequencing effort (not shown) was predicted by inverting the selected function for x such that the value of the function was 0.9 times the estimated sample richness. Note that for certain 3 and 4 parameter functions the R2 values are extremely high and differ only in the fourth or fifth decimal places (R2>0.999) yet the estimated richness can differ substantially (cf. Power Michaelis Menten (2) vs. Weibull Cumulative). For such functions, the AICc values also tend to differ by relatively large amounts, such that a model averaging strategy based on AIC weights would differ little from simply choosing the lowest-AICc model (Burnham and Anderson, 2002), and any assessment of model selection uncertainty based on AIC-weights is unlikely to predict the level of selection uncertainty observed in simulations (see Table S3). This latter is likely the result of the neglected error correlation in the functional fits.

Function	Formula (x = #reads-1)	Number of Parameters	R^2	AICc	Ŝ
Michaelis Menten	(ax)/(b+x)+1	2	0.98414	4976	1520
Negative Exponential	a(1-exp(-bx))+1	2	0.93681	5670	1317
Power Michaelis Menten (1)	$ax^c/(b+x^c)+1$	3	0.99977	2856	1927
Power Michaelis Menten (2)	$ax^c/(b+x)^c+1$	3	0.99995	2086	1679
Power Negative Exponential	$a(1-exp(-bx))^c+1$	3	0.99947	3274	1459
Weibull Cumulative	$a(1-exp(-bx)^c)$	3	0.99992	2323	1568
Michaelis Menten + offset	(ax)/(b+x)+1+c	3	0.99680	4174	1590
Negative Exponential + offset	a(1-exp(-bx))+1+c	3	0.98639	4901	1373
Power Michaelis Menten (1) + offset	$ax^c/(b+x^c)+1+d$	4	0.99988	2545	1864
Power Michaelis Menten (2) + offset	$ax^c/(b+x)^c+1+d$	4	0.99995	2088	1679
Power Negative Exponential	$a(1-exp(-bx))^c+1+d$	4	0.99957	3169	1467
Weibull Cumulative + offset	$a(1-exp(-bx)^c)+d$	4	0.99992	2316	1565

Table C. Semiparametric functional fits to bottom sample collector's curve data and corresponding estimates of total species richness. A set of 12 convex, saturating functions were fitted to the rarefied species accumulation curve, sampled at intervals of 1000 reads (hence 576 data points), using the nonlinear least squares function "nls" in R to estimate the parameters a, b etc. The absolute quality of the fits was measured using the generalized R2 values (defined for nonlinear fit as 1 - RSS/SSM, where RSS is the residual sum of squares and SSM is the sum of squares of the sample mean). The best-approximating model was selected as that which minimized Akaike's Information Criterion (AICc, in this case the Power Michaelis Menten (2) + offset function was selected). The selected model was then used to estimate the total sample richness S as the asymptotic value of the function at x = Inf (final column shows the estimates for all candidate functions). Required sequencing effort (not shown) was predicted by inverting the selected function for x such that the value of the function was 0.9 times the estimated sample richness. Note that for certain 3 and 4 parameter functions the R2 values are extremely high and differ only in the fourth or fifth decimal places (R2>0.999) yet the estimated richnesses can differ substantially (cf. Power Michaelis Menten (2) vs. Weibull Cumulative). For such functions, the AICc values also tend to differ by relatively large amounts, such that a model averaging strategy based on AIC weights would differ little from simply choosing the lowest-AICc model (Burnham and Anderson, 2002), and any assessment of model selection uncertainty based on AIC-weights is unlikely to predict the level of selection uncertainty observed in simulations (see Table S3). This latter is likely the result of the neglected error correlation in the functional fits.

Function	Formula	Number of Parameters	R^2	AICc	$\hat{S}$
Michaelis Menten	$(ax)/(b+x)+1$	2	0.98873	6899	4947
Negative Exponential	$a(1-\exp(-bx))+1$	2	0.95179	7737	4224
Power Michaelis Menten (1)	$ax^c/(b+x^c)+1$	3	0.99986	4380	6122
Power Michaelis Menten (2)	$ax^c/(b+x)^c+1$	3	0.99999	2897	5425
Power Negative Exponential	$a(1-\exp(-bx))^c+1$	3	0.99959	4996	4666
Weibull Cumulative	$a(1-\exp(-bx)^c)$	3	0.99999	3062	4981
Michaelis Menten + offset	$(ax)/(b+x)+1+c$	3	0.99758	6014	5157
Negative Exponential + offset	$a(1-\exp(-bx))+1+c$	3	0.98893	6891	4397
Power Michaelis Menten (1) + offset	$ax^c/(b+x^c)+1+d$	4	0.99992	4081	5971
Power Michaelis Menten (2) + offset	$ax^c/(b+x)^c+1+d$	4	0.99999	2566	5435
Power Negative Exponential	$a(1-\exp(-bx))^c+1+d$	4	0.99974	4729	4702
Weibull Cumulative + offset	$a(1-\exp(-bx)^c)+d$	4	0.99999	2924	4996

Table D. Simulation-based tests of estimator performance, considering estimates of both the total species richness (S) and the required sequencing effort (RSE) i.e. number of final reads required to observe a given fraction of the total richness in a new sample (e.g. 0.7S means 70% of the total richness). For each of four parametric distributions (Table D1) Poisson log-normal, Poisson log-student and Table D2 Poisson inverse-Gaussian, and Sichel) an ensemble of 80 sets of community abundances were randomly sampled from the parametric distribution; species data were then simulated by sampling from multinomial distributions with probabilities defined by the community abundances for each ensemble member. Distribution parameter values, including the total species richness, were also varied between ensemble members by sampling from the posterior distributions fitted to the observed data. Estimator performance is summarized by the %BIAS (ensemble average of estimate minus true value) and %RMSE (root-mean-square error), normalizing by the ensemble mean of the true value in both cases. Non-parametric species richness estimators included the Chao1 lower bound estimate (Chao, 1984), the coverage-based estimator for highly heterogeneous communities (ACE-1; Chao and Lee, 1992; Chao *et al*., 2000) and the bias-corrected Chao estimate iChao (Chiu *et al*., 2014). The ACE-1 estimator was tested using two values of the cut-off count k to define "rare" species: the default value k = 10 and a larger value k = 100 as recommended by Chao and Shen (2012) for microbial communities (note, the estimated CV of the "rare" species was < 0.8 for k = 10 but > 0.8 for k = 100, where 0.8 is a threshold above which Chao and Shen (2012) recommend ACE-1 in preference to ACE). RSE was estimated for each nonparametric estimator by inverting the expression in Table 1 of Chao *et al*. (2014) and substituting the corresponding estimates of the zero-count

frequency f0 = (S - S_{obs}) (using ACE-1 this is identical to the method proposed in Chao and Shen (2012) based on Shen *et al*. (2003) except for a negligible bias correction). Similar results (not shown) were obtained by substituting into equation (12) in Chao *et al*. (2009) (see also Colwell *et al*., 2012, equation 11). A semi-parametric AICc-selected estimator SP (AICc) was constructed by fitting 12 different functions to the collector's curves (rarefied species richness vs. sampling effort) and choosing the function with the lowest Akaike's Information Criterion (AICc). Total richness was then estimated as the asymptotic value of the selected function (see Table S2), and RSE was estimated by inverting the selected function for sampling effort given the required fraction of asymptotic richness. Nonparametric estimates were calculated using the R package SPECIES (Wang, 2011) and semiparametric functions were fitted using the nonlinear least squares function "nls" in R (R Core Team, 2013).

Table D1

Estimator	Sample	S(lognormal)		RSE(0.7S, lognormal)		S(logstudent)		RSE(0.8S, logstudent)	
		%BIAS	%RMSE	%BIAS	%RMSE	%BIAS	%RMSE	%BIAS	%RMSE
Chao	Surface	-25.8	26.8	-86.2	116.1	-22.1	22.9	-76.2	90.1
ACE-1 (k=10)	Surface	-24.5	25.4	-85.5	114.4	-18.9	19.4	-71.3	84.0
ACE-1 (k=100)	Surface	0.9	4.3	-21.0	54.4	50.2	60.0	95.2	113.4
iChao	Surface	-23.4	24.5	-84.8	114.1	-19.1	19.9	-72.1	85.9
SP(AICc)	Surface	-1.0	4.5	-6.2	33.3	-2.4	11.6	-2.8	70.3
Chao	Bottom	-14.9	15.1	-70.1	71.9	-24.3	24.8	-73.9	81.3
ACE-1	Bottom	-14.2	14.4	-70.8	72.4	-18.8	19.0	-66.2	72.6
ACE-1 (k=100)	Bottom	7.5	8.1	54.1	55.2	93.8	101.5	167.0	178.3
iChao	Bottom	-12.7	12.9	-71.1	72.5	-20.6	21.0	-69.0	76.4
SP(AICc)	Bottom	3.7	4.1	23.3	27.5	3.6	10.0	31.3	60.8

Table D2

Estimator	Sample	S(inverse Gaussian)		RSE (0.9S, inverse Gaussian)		S(Sichel)		RSE(0.9S, Sichel)	
		%BIAS	%RMSE	%BIAS	%RMSE	%BIAS	%RMSE	%BIAS	%RMSE
Chao	Surface	-5.7	6.6	-36.6	49.6	-6.3	7.8	-38.3	53.9
ACE-1 (k=10)	Surface	-3.5	4.2	-26.9	36.6	-3.7	4.9	-27.5	40.3
ACE-1 (k=100)	Surface	40.6	45.9	283.0	313.4	53.2	60.5	323.0	361.5
iChao	Surface	-3.3	4.3	-25.9	39.2	-3.6	5.5	-27.5	43.8
SP(AICc)	Surface	1.4	6.8	81.6	448.9	4.1	12.2	237.9	921.5
Chao	Bottom	-5.9	6.0	-33.5	34.9	-7.4	7.9	-38.5	44.1
ACE-1	Bottom	-3.7	3.8	-23.8	25.0	-3.6	3.9	-24.7	28.5
ACE-1 (k=100)	Bottom	43.5	44.3	306.2	309.8	76.0	81.2	396.9	416.9
iChao	Bottom	-3.2	3.5	-21.6	23.3	-4.2	4.8	-27.2	33.0
SP(AICc)	Bottom	1.3	7.1	51.6	206.8	3.1	12.5	138.5	391.6
